# Cardiovascular Epidemiological Research in China: A Wake-up Call No One Can Afford to Ignore

**DOI:** 10.1016/j.lanwpc.2021.100308

**Published:** 2021-10-21

**Authors:** Xiang Wei, Ding-Sheng Jiang, Song Wan

**Affiliations:** 1 Division of Cardiothoracic and Vascular Surgery, Sino-Swiss Heart-Lung Transplantation Institute, Tongji Hospital, Tongji Medical College, Huazhong University of Science and Technology, Wuhan, Hubei, China; 2 Key Laboratory of Organ Transplantation; Ministry of Education; NHC Key Laboratory of Organ Transplantation; Key Laboratory of Organ Transplantation; Chinese Academy of Medical Sciences, Wuhan, Hubei, China; 3 Division of Cardiothoracic Surgery, Department of Surgery, The Chinese University of Hong Kong, Prince of Wales Hospital, Hong Kong, China

**Keywords:** Aortic dissection, hypertension, preventive medicine

One of the most elementary principles in life is that “you cannot improve what you don't measure.” In this regard, Tang and colleagues [1] are to be commended for achieving an important benchmark in cardiovascular epidemiological research. Employing the Urban Employee Basic Medical Insurance (UEBMI) database in China, these investigators examined the incidence and 3-year mortality rates of aortic dissection among almost 347 million residents in 23 provinces (i.e., covering more than 52% of the entire urban population of China). They revealed the age- and sex-adjusted incidence of aortic dissection to be 2.78 per 100,000 person-years in urban China, whereas the subsequent overall 3-year survival rate was 83.7%. [1] Moreover, their findings confirmed that the geographic disparities of such an incidence were consistent with the prevalence of hypertension in China. [1] As a vital part of the National Insurance Claims for Epidemiological Research (NICER) study, the current project obviously carries significant epidemiological and clinical implications.

First, the reported incidence of aortic dissection in China could just be the tip of iceberg; the real-world data should be taken more seriously. As Tang and colleagues [1] correctly highlighted, owing to the nature of the study design, many out-of-hospital deaths due to aortic dissection were not captured by the UEBMI database using proper clinical diagnoses (not to mention those from the rural areas in China are likely having even greater incidence of this disease with higher associated mortality). This inevitably resulted in an underestimation of aortic dissection incidence and overestimation of the survival rates in the current study. [1] In fact, most cardiac surgeons in China have clearly experienced an increasing trend of aortic dissection over the past decade. For example, the numbers of surgical operations performed for diseases of the thoracic aorta in China were 15,593 in 2016, 19,585 in 2017, 22,898 in 2018, and 26,967 in 2019. [2,3] Over the past 7 years, the annual increasing rate has constantly approached 20%. [3] It is estimated that about half to approximately two-thirds of these operations were related to aortic dissection (including both Stanford types A and B). In 2019, for the first time ever, at least one out of 10 cardiac surgical operations in China was related to the disease of the thoracic aorta. [3]

The second but more alarming fact is that compared to reported cases in Western countries, physicians in China are dealing with considerably younger patients with aortic dissection. Meanwhile, many of these patients were not even aware of their pre-existing comorbidities such as hypertension. As shown by Tang and colleagues [1], the mean age of the patients presented with aortic dissection was only 58.8 years. It is noteworthy that a majority (77.1%) of these patients were male, with a high crude incidence of aortic dissection (4.97% per 100,000 person-years), while only 29% of these men were diagnosed with hypertension before the onset of aortic dissection. [1] Considering the studied patient sub-population were all urban residents in a relatively better healthcare environment, it is understandable that the challenges associated with the diagnosis and treatment of aortic dissection for those who live in rural areas could be substantially greater. Conversely, the higher incidence of aortic dissection among elderly patients (e.g., > 11.5% per 100,000 person-years among those aged 70 years and above in the current study [1]) represents another significant healthcare burden in China, since the aging trend in Chinese society is indisputable. According to the latest national population survey [4], there were more than 190 million Chinese citizens aged ≥ 65 years, representing 13.5% of the entire population in the country (as of November 2020). It is predicted that this elderly subgroup may surge to over 300 million within 15 years (i.e., representing > 20% of the national population in 2036). Among Chinese citizens aged ≥ 75 years, however, recent evidence indicates that 59.8% of them were diagnosed with hypertension. [5]

It was no surprise to find that the incidence of aortic dissection was significantly higher in Northwest China than in South China, which highly correlates with the prevalence of hypertension in China. [1,5] Apart from the potential impact of different climate environments, the variation in diet, lifestyle, and genetic background across Chinese ethnic groups may also play important roles in inducing such a difference. As far as the development of aortic dissection is concerned, hypertension is undoubtedly the most crucial risk factor. There are more than 245 million patients with hypertension in China. [5] Prevention and proper nationwide control of this disease definitely represents one of the top healthcare priorities in the country. Despite the expected difficult and slow progress, ongoing research in this field has offered some hope with encouraging early clinical outcomes. For instance, a recent prospective, multicentre, randomized Strategy of Blood Pressure Intervention in the Elderly Hypertensive Patients (STEP) trial from China, involving 8511 elderly patients with hypertension, confirmed that intensive treatment with a systolic blood-pressure target of 110-130 mmHg resulted in a lower incidence of cardiovascular events than standard treatment with a target of 130-150 mmHg. [6]

So, where should we head from here? The Chinese word for crisis is composed of two characters, one meaning “danger” and the other “opportunity”. Indeed, while we have been alerted to the crisis by the updated data, [1] our interventions can also be inspired by ancient wisdom. A popular quote from a seminal book in traditional Chinese medicine, *Yellow Emperor's Inner Classic* (206 BCE–220 CE), reminds us, “the superior doctor prevents illness, the inferior doctor treats actual disease”. Even though being cardiologists or cardiothoracic surgeons most of us have to continually treat actual disease, the importance of preventive measures for hypertension at large and for aortic dissection in particular can never be overemphasized, especially in a country that is home to almost one-fifth of the Earth's population.

## Author contributions

XW: designed and critically reviewed the commentary

DSJ: designed and critically reviewed the commentary

SW: designed and wrote the commentary

## Declaration of Competing Interest

We declare that we have no conflicts of interest or financial disclosures.
